# Optimization in the Context of COVID-19 Prediction and Control: A Literature Review

**DOI:** 10.1109/ACCESS.2021.3113812

**Published:** 2021-09-17

**Authors:** Elizabeth Jordan, Delia E. Shin, Surbhi Leekha, Shapour Azarm

**Affiliations:** Department of Mechanical EngineeringUniversity of Maryland College Park MD 20742 USA; Department of Epidemiology and Public HealthUniversity of Maryland School of Medicine 12264 Baltimore MD 21201 USA

**Keywords:** Optimization, COVID-19, decision support, screening testing, prediction, prevention, control, resource allocation, vaccination, literature review

## Abstract

This paper presents an overview of some key results from a body of optimization studies that are specifically related to COVID-19, as reported in the literature during 2020-2021. As shown in this paper, optimization studies in the context of COVID-19 have been used for many aspects of the pandemic. From these studies, it is observed that since COVID-19 is a multifaceted problem, it cannot be studied from a single perspective or framework, and neither can the related optimization models. Four new and different frameworks are proposed that capture the essence of analyzing COVID-19 (or any pandemic for that matter) and the relevant optimization models. These are: (i) microscale vs. macroscale perspective; (ii) early stages vs. later stages perspective; (iii) aspects with direct vs. indirect relationship to COVID-19; and (iv) compartmentalized perspective. To limit the scope of the review, only optimization studies related to the prediction and control of COVID-19 are considered (public health focused), and which utilize formal optimization techniques or machine learning approaches. In this context and to the best of our knowledge, this survey paper is the first in the literature with a focus on the prediction and control related optimization studies. These studies include optimization of screening testing strategies, prediction, prevention and control, resource management, vaccination prioritization, and decision support tools. Upon reviewing the literature, this paper identifies current gaps and major challenges that hinder the closure of these gaps and provides some insights into future research directions.

## I. Introduction

In late 2019, a Chinese ophthalmologist alerted fellow doctors in Wuhan, China, of an alarming, new virus [1]. By March 2020, COVID-19, a highly contagious, airborne coronavirus, had ravaged nearly every continent in the world, causing the World Health Organization (WHO) to declare a global pandemic [2].

The sudden rapid outbreak of the coronavirus has posed significant challenges to all communities. Due to the novel nature of COVID-19 and its emerging new strains, researchers and doctors have struggled to fully understand all variables attributed to the virus, such as its transmission rate, making it extremely difficult to accurately model [3]. Even as more information and data are becoming available, these variables continue to be dynamic, which makes it difficult to quantify them. During the beginning of the pandemic, the lack of good models and sufficient data on confirmed cases and deaths also made it challenging to predict future cases and plan for critical hospital resource demands and appropriate mitigation strategies. Due to the unfamiliarity and novelty of the coronavirus, mixed messages were sent regarding the potential severity of the outbreak, the importance of mask wearing and social distancing, and other related measures, leading to ad hoc, unplanned lockdowns, and spikes in COVID- 19 cases. As doctors and scientists were beginning to better understand the destructive and disruptive nature of COVID-19, supply chains and hospitals around the world were unable to keep up with the demand for ventilators, personal protective equipment (PPE), and intensive care unit (ICU) beds, causing critically ill patients to be turned away [4]. Some hospitals began to construct makeshift ICU wards in parking lots and garages to accommodate the influx and vast surge in patients needing critical care due to the effects of COVID-19 [5].

These challenges required quick solutions and responses and presented many opportunities for optimization. Examples include optimizing prediction models to quantify future COVID-19 cases [6]–[24], optimizing the allocation of critical hospital supplies to treat COVID-19 patients [25]–[32], and developing decision support tools to guide data-driven mitigation strategies to combat the spread of the virus [33]–[37]. While numerous studies have tried to tackle these challenges, the COVID-19 pandemic continues to expose the flaws and shortcomings of current systemic policies, many of which were unanticipated and unprepared for. Analyzing available published literature on COVID-19 from multi-disciplinary fields and various countries reveals which areas, whether resource allocation or non-pharmaceutical interventions (such as mask mandates or social distancing), need to be addressed to sufficiently contain the pandemic. In addition, should another pandemic like COVID-19 ravage the globe in the future, the current shortcomings will need to be addressed to minimize deaths and hospitalizations while maximizing containment of the virus. Government response time was also seen as a catalyst or barrier to containing the spread of the virus. For example, in New Zealand, the government immediately closed all borders in conjunction with lockdowns and mask mandates. However, in the United States, where the government was much more hesitant, reluctant, or unable to dictate a shutdown with mask mandates, death tolls reached nearly 600,000, in comparison to 26 deaths in New Zealand due to COVID-19 (as of June 15, 2021). Although population density is a contributing factor and directly parallels the number of deaths, the United States, unfortunately, witnessed the inability of some hospitals to accept patients due to ICU shortages, whereas New Zealand remained constant.

In this paper, our aim is to present a review of the literature pertaining to formal optimization studies related to the prediction and control of the COVID-19 pandemic. These studies include optimization of screening testing strategies, prediction models, resource allocation, vaccine distribution, and mitigation policies to curb the spread, as well as decision support tools. From these studies, it is observed that since COVID-19 is a multifaceted problem, it cannot be studied from a single perspective or framework. Thus, four new and different frameworks are proposed that capture the essence of analyzing COVID-19. We believe it would be of value to present the reader with these frameworks that provide a holistic view of COVID-19 and its contributing aspects. These frameworks may even enable better preparedness for future pandemics by helping the reader visualize the structure and connections between the various aspects related to the virus. The frameworks can also provide insight into which aspects of the pandemic should be focused on and the relationships between various optimization models pertaining to the pandemic.

This paper provides the following contributions to the existing literature: (i) a review of key studies in the area of optimization in the context of COVID-19 prediction and control; (ii) four new frameworks to gain a holistic view of COVID-19, or future pandemics; (iii) identification of current gaps and challenges; and (iv) insight into future research directions.

The remainder of this paper is organized as follows. Section II discusses the literature search approach and the criteria used for consideration of the reviewed papers. Section III provides a broad overview of papers that fit into the realm of optimization and COVID-19 in relation to prediction and control and mentions current efforts related to tracking the virus transmission and the developed decision support tools. Section IV describes the four new frameworks and reviews the literature related to each specified topic. Finally, Section V summarizes the identified gaps, challenges, and lessons learned, followed by concluding remarks in Section VI.

## II. Literature Search Approach

The main source used for the literature search was Web of Science (WoS), which includes a rich collection of various databases that are referenced in both the scientific, engineering, and medical communities. Google Scholar was an additional source we utilized to ensure that we covered a broader horizon and did not potentially miss any significant publications. The papers considered for the literature search were selected from those published between December 2019 and June 2021. The two main keywords used for the search were the words *optimization* and *COVID-19*. More specific keywords were also used, such as prevent*, *testing*, vaccin*, predict*, forecast*, resource, allocat*, distribut*, mitigat*, and *decision support*. The symbol “*” is used as a wildcard character and can be substituted by any number of alphabetical letters that can complete the incomplete word, e.g., “vaccin*” can include vaccin*e*, vaccin*ating*, vaccin*ation*. The more specific keywords were chosen based on general knowledge related to pandemic response and preparedness [38] and based on the scope of this review.

**FIGURE 1.**
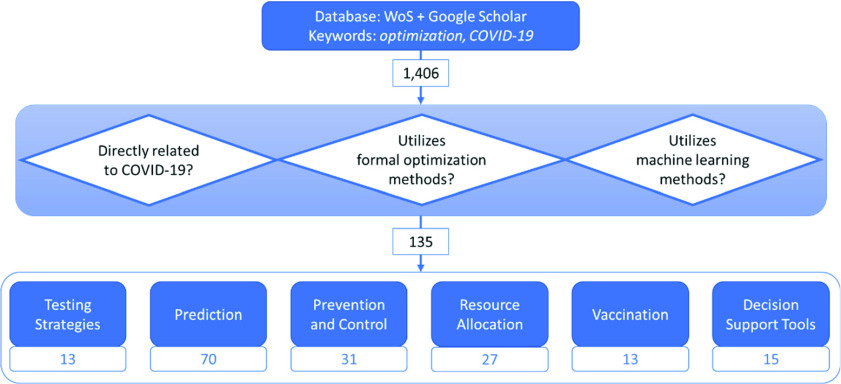
Flowchart of literature selection process. The final selected papers were categorized by the respective topics shown.

This literature review covers the papers which we believe are the key and unique papers in the area of optimization studies related to the public health aspect of COVID-19, particularly those that fall under the umbrella topics of prediction and control. Fig. 1 shows the high-level process of the downselection approach of the papers considered. The specific selection criteria used to downselect will be discussed below. It is worth mentioning that some papers fit and thus are counted under multiple categories. For example, a paper may fall into both the “Prediction” and “Prevention and Control” categories, or into both the “Resource Allocation” and “Vaccination” categories and counted as such in both categories.

As shown in Fig. 1, we considered studies related to the optimization of COVID-19 testing strategies, prediction models of cases and deaths due to the virus, forecasting the demand for critical hospital supplies to treat patients, determination of optimal pandemic mitigation strategies, resource allocation, vaccine distribution, as well as development of decision support tools to aid in combating the spread.

In this review, we have included studies that used *formal optimization methods*, *machine learning techniques* (where optimization algorithms were explicitly used in the process), and studies that were *directly related to COVID-19*. By *formal optimization methods*, we mean that the authors of the reviewed paper identified a problem that had emerged as a result of COVID-19 and formulated that problem in mathematical or simulation forms. This is then followed by setting up an optimization problem by defining decision variables or strategies (such as the number of ventilators to allocate to a specific region), a specific objective function(s) to minimize or maximize (such as minimizing the number of deaths due to COVID-19), and a set of constraints or requirements (such as capacity or resource limits) to satisfy. Subsequently, this optimization problem was solved using optimization techniques, see, e.g., [39]–[41]. Since *machine learning* [42]–[45] has become prevalent in many applications, we also considered papers that applied optimization techniques in the context of machine learning models to estimate or optimize certain parameters. Overall, we focused on papers in which the optimization problem was *directly related to COVID-19*- e.g., papers related to the management of the virus, rather than papers addressing the effects of the pandemic on various aspects of our lives, as discussed next.

For clarification, we did not consider papers that fell into the following two categories. The first category included what we call *informal optimization* papers, which did not utilize formal optimization methods or techniques. Many studies used the term “optimization” loosely, or informally – e.g., there was no formal formulation of an optimization problem. Instead, an “optimal” solution was reported by selecting an option that yielded the “best” results from a small set of known, pre-selected alternatives (see [46]–[48]). Other papers focused on the optimization of certain procedures or protocols, thus establishing a set of “best practices” (see [49]–[52]). The second category consisted of papers *indirectly related to COVID-19*. This category of papers covered the optimization of certain aspects that were not directly related to COVID-19, but were rather a representation of the *effects* of COVID-19 on different domains. Examples of the second category include disruptions in supply chains (e.g., rise in new and used car prices due to chip shortages), electricity market (e.g., significant increase in residential electricity demand vs. substantial decrease in commercial demand), or transportation sector (e.g., fluctuations in fuel costs), see, for example, [53]–[55].

## III. Broad Perspective of the COVID-19 Optimization Literature

**FIGURE 2.**
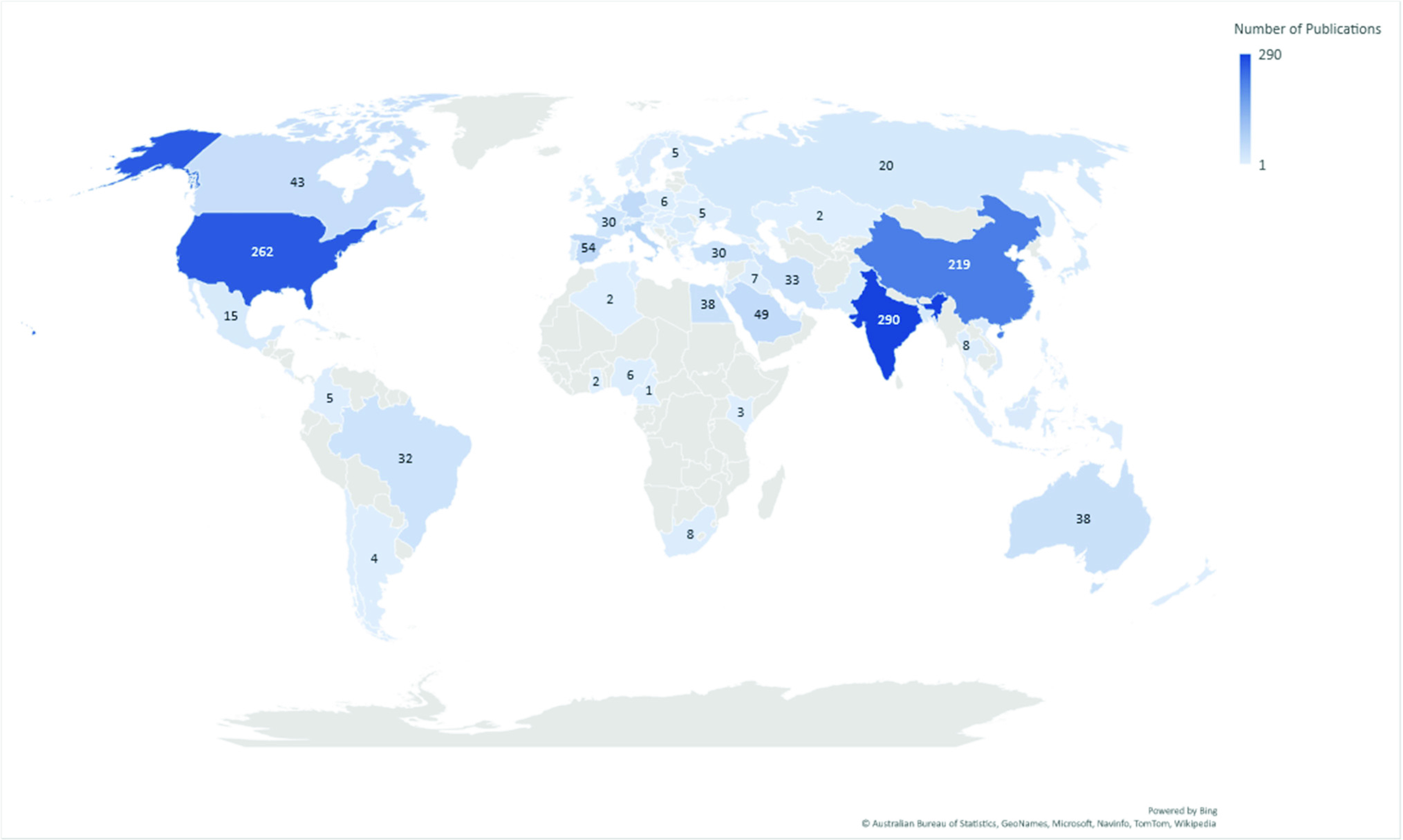
Number of publications by country (using the keywords optimization and COVID-19).

A quick search of all the databases on WoS revealed that as of June 30, 2021, over a year after the start of the pandemic, countries all over the world had studied COVID-19 through the lens of optimization. Fig. 2 shows a density map of papers published by country with the keywords *optimization* and *COVID-19*. India, the United States, and China are the top three leaders for the number of papers, with 290, 262, and 219 publications, respectively. The remaining countries have up to 60 publications on the topic of *optimization* and *COVID-19*.

**FIGURE 3.**
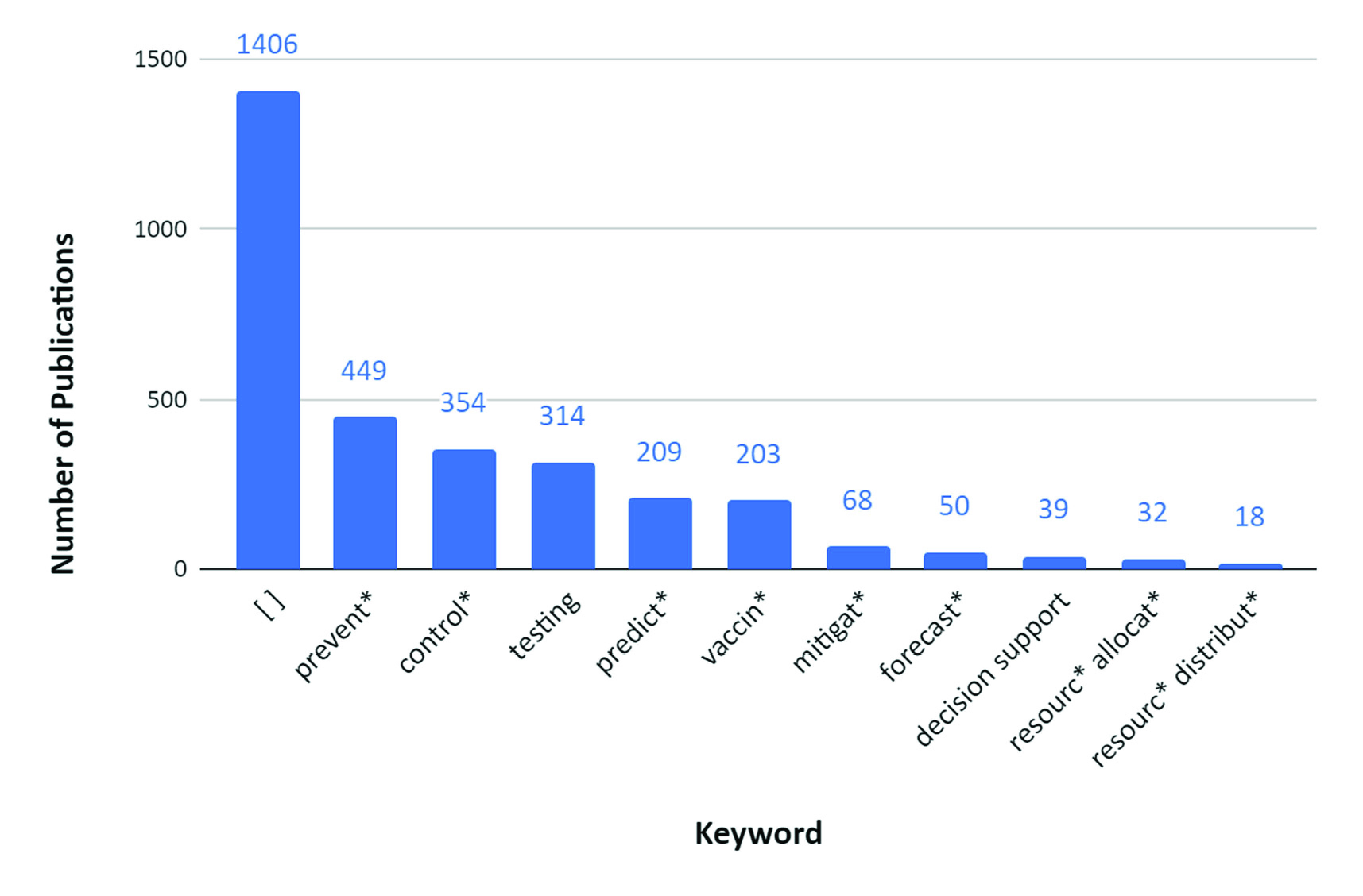
Publications on the topic of “optimization and COVID-19” with additional keywords.

It was also noted that the majority of the literature related to optimization and COVID-19 focused on topics related to the prediction (i.e., forecasting of cases), detection methods, testing, and prevention of the virus, while less attention was given to topics concerning decision support tools and allocation or distribution of resources (Fig. 3). Fig. 3 illustrates the number of publications grouped by specific keywords. The blue bars represent the presence of the two main keywords *optimization* and *COVID-19*, and an additional, more specific keyword (e.g., *testing*, predict*, prevent*). For example, there were 203 papers that resulted from searching the keywords “optimization, covid-19, vaccin*” in the topic across all the databases in WoS. It is important to keep in mind that a paper can fall into several categories. For example, a study on the optimization of vaccine distribution may belong to both the vaccin* and resourc* distribut* categories.

With a plethora of papers published on various topics in relation to COVID-19, some literature reviews emerged that grouped similar papers and provided an overview of the current state-of-the-art in a particular topic and suggested future directions of research. Some of the most common literature review topics included: general overviews of the COVID-19 pandemic [56]–[58], COVID-19 and its relation to or effect on other health issues [59]–[61], diagnosis and treatment methods [62]–[66], and effects of the pandemic on various areas of our life (e.g., supply chain [67]–[69] or the economy [70], [71]). However, there has yet to be a review of the literature which focuses on the optimization studies in relation to the COVID-19 pandemic, specifically pertaining to prediction and control – this paper closes that gap.

In addition to studies and publications, the development of informative COVID-19 dashboards and prediction tools [72]–[74] has been approached by many scientists and researchers. For example, the Johns Hopkins University (JHU) COVID-19 dashboard has tracked the transmission of the virus both locally, in the US, and globally, including the number of cases, deaths, and hospitalizations, since the start of the pandemic. The European Centre for Disease Prevention and Control also reports the number of cases and deaths globally. Many university labs (e.g., MIT, UT Austin), news sources (such as the Washington Post, New York Times), and organizations (CDC, The COVID Tracking Project, CovidActNow) have also created COVID-19 trackers to visualize the spread and impact of the virus. In addition to the number of different cases, some of these dashboards also provide demographic data (JHU, The COVID Tracking Project). In addition to trackers, researchers and scientists have also focused on developing interactive prediction and forecasting models to aid in determining appropriate intervention policies (e.g., lockdowns, social distancing) and hospital resource allocation, including a couple of the well-known models developed by IHME at the University of Washington and the CDC. Table 1 summarizes some examples of such tools and their capabilities. Additional information and examples of various tools and resources pertaining to modeling the pandemic can be found in MIDAS,^1^ the National Institutes of Health,^2^ and the American Hospital Association^3^ repositories.^1^https://midasnetwork.us/covid-19/^2^https://datascience.nih.gov/covid-19-open-access-resources^3^https://www.aha.org/guidesreports/2020-04-09-compendium-models-predict-spread-covid-19

**TABLE 1:** Example of COVID-19 Tools

Tool Name	Source	Tool Type	Description
COVID-19 Dashboard	John Hopkins University (JHU)	Dashboard^*^	Tracks # of cases, deaths, testing rate, vaccination status globally and within the USA
COVID-19 Situation Dashboard COVID-19 Vaccine Tracker	European Center for Disease Prevention and Control	Dashboard	Tracks # of cases, deaths, and vaccinations in reporting European countries
COVID-19 Indoor Safety Guideline	Massachusetts Institute of Technology (MIT)	Model^**^	Predicts time under certain circumstances to contract COVID-19 if infected individual is in close proximity
Localized COVID-19 Model and Scenario Planner	Qventus	Model	Localized (by hospital) estimates of COVID-19 cases and hospital resource needs. The user has the ability to modify a variety of model parameters
Washington Post COVID-19 Tracker	Washington Post	Dashboard	Tracks # of cases, deaths, tests, hospitalizations, and vaccinations in the United States
New York Times COVID-19 Tracker	New York Times	Dashboard	Tracks # of new reported cases, deaths, vaccinations, and regions with high positivity rates globally
COVID Data Tracker	Centers for Disease Control and Prevention (CDC)	Dashboard	Tracks # of cases, deaths, testing, vaccination, and hospitalizations, as well as demographic data
The COVID Tracking Project	The COVID Tracking Project	Dashboard	Tracks # of cases, deaths, hospitalizations, daily tests administered, and key metrics by state in the USA
U.S. COVID Risk & Vaccine Tracker	CovidActNow	Dashboard	Tracks # of vaccinations, risks by region, infection and positivity rates, and vulnerability levels
COVID-19 Projections	Institute for Health Metrics and Evaluation (IHME)	Model	Predicts # of deaths, hospital resource use, infections and testing, and effects of masking and social distancing

^*^ Dashboards are what we call tools that merely present a visualization of the current state of the pandemic; these are data visualization tools

^**^ Models are what we call tools that use existing data to provide more insight - be it the prediction of future cases or hospital demand; models use data and epidemiological/optimization/machine learning/etc. models to generate new information

The next section will discuss the four proposed frameworks and summarize the literature reviewed for this paper.

## IV. The Four Frameworks and Literature Review

In this section, we propose four different frameworks that capture the essence of analyzing the approach to studying COVID-19 and related optimization models. We follow up with a more detailed review of the optimization studies in the context of prediction and control. The four frameworks are: (i) microscale vs. macroscale perspective; (ii) early stages vs. later stages perspective; (iii) aspects with direct vs. indirect relationship to COVID-19; and (iv) compartmentalized perspective. These frameworks can provide some insights into the structure and relationships between the various aspects of the pandemic. Consequently, this can also shed light on the connections that can be made among the corresponding optimization models.

**FIGURE 4.**
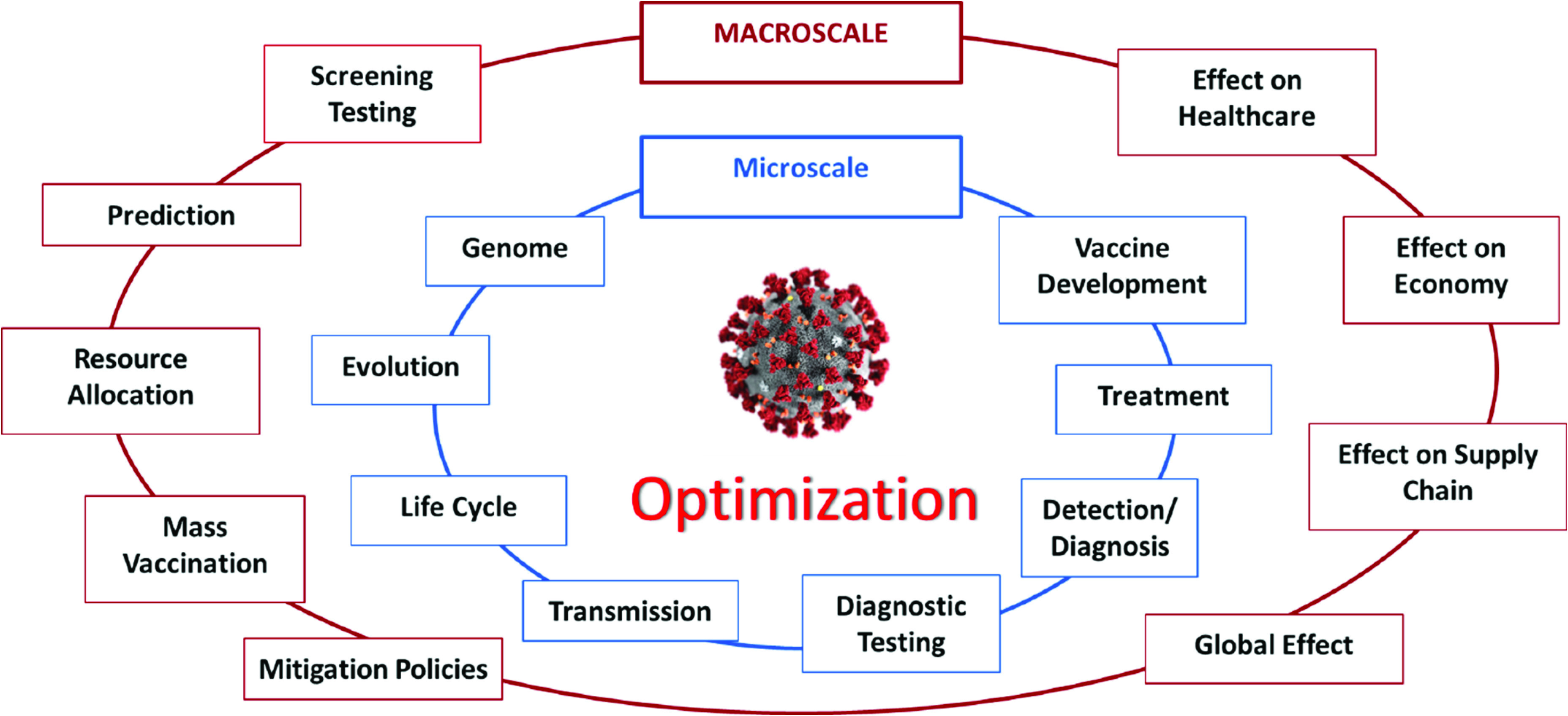
Macroscale vs microscale perspective framework.

**FIGURE 5.**
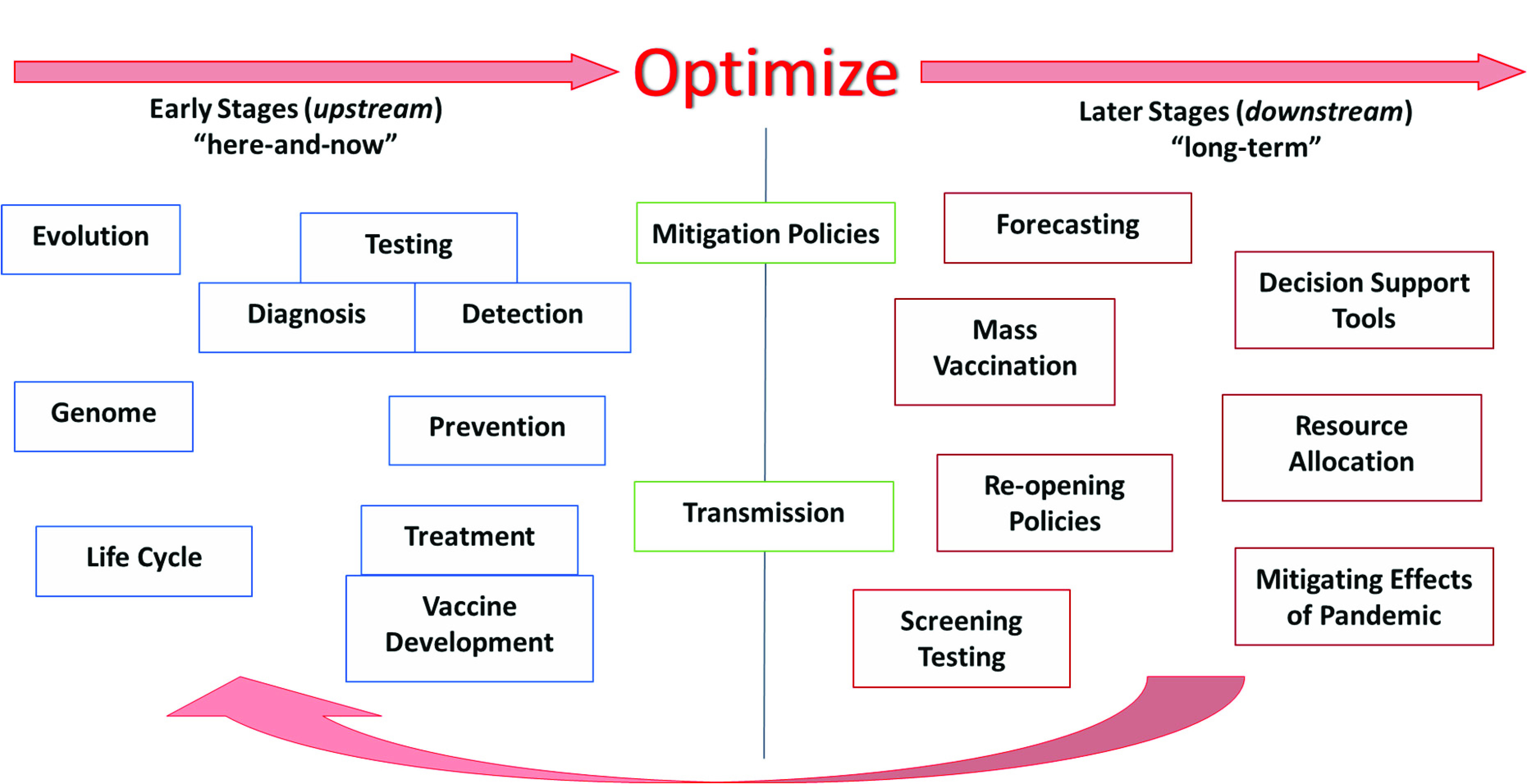
Early stages vs later stages perspective framework.

**FIGURE 6.**
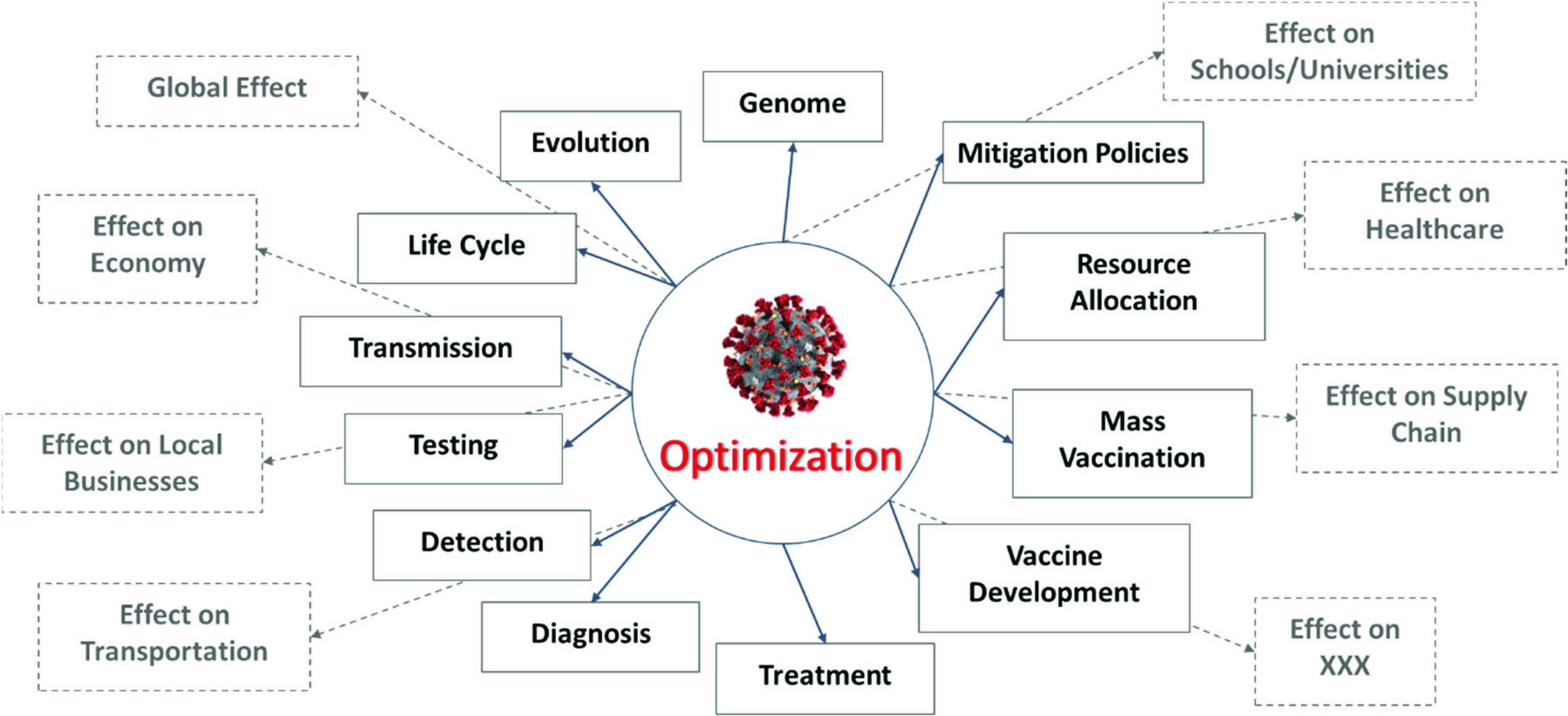
Direct vs indirect relationship to COVID-19 framework.

**FIGURE 7.**
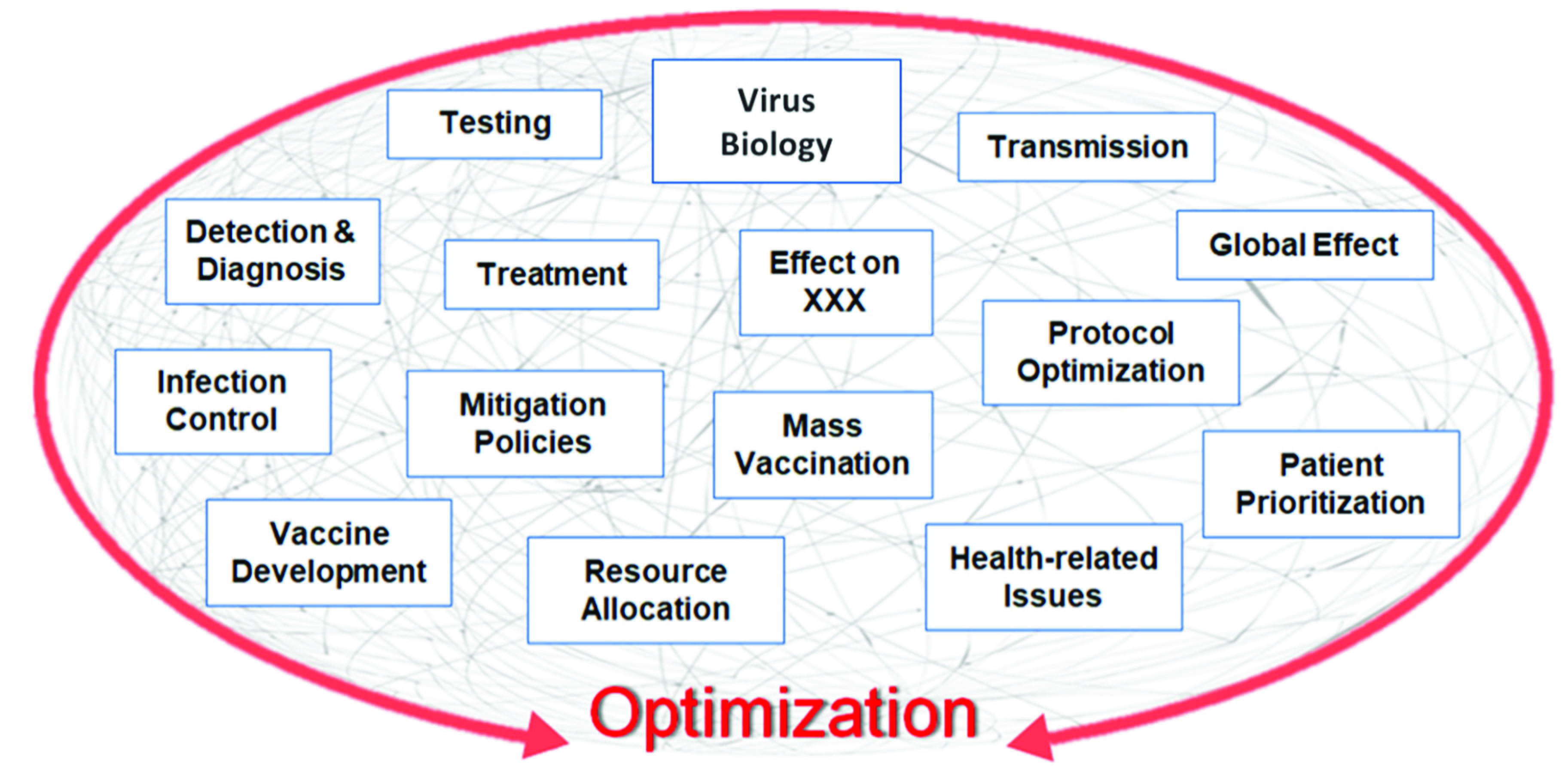
Compartmentalized perspective framework.

(i) *Microscale vs. Macroscale Perspective*. A virus is microscopic, yet has a macroscopic effect on our lives. Therefore, a virus can be studied on a *microscale level* and (or all the way to) a *macroscale level* (Fig. 4), or, alternatively, from a *medical* vs. *public health* perspective, where the *medicine* focus is on the *individual*, whereas the *public health* focus is on the *population* [75]. At the *microscale level*, the virus is studied in relation to itself and how it transmits to and affects an individual person. For example, studies of the genome of the virus, or its evolution and transmission mechanisms; the development of optimal testing, diagnostic, and detection methods to identify the virus in infected patients; the identification of the “best” ways to treat the virus, including therapies, drugs, and vaccines - are categories that would fall into the *microscale level* of analysis. At the *macroscale level*, the virus is studied in relation to how it spreads within a population, how it can be controlled within a population via policies or mitigation strategies, how it affects healthcare, the economy, and other aspects of our life. Optimization can play an important role in all of these studies. For example, optimal strategies that minimize transmission dynamics of the virus, improve forecasting of the demand for critical hospital supplies, minimize the detrimental effects of COVID-19 in a certain area, and suggest optimal recourse strategies to mitigate the ramifications - are categories that would fall into the *macroscale level* of analysis.
(ii) *Early stages vs. Later stages Perspective*. From a temporal perspective, the evolvement of the pandemic can be divided into two sections: the early stage and later stage, or alternatively, upstream and downstream (Fig. 5). The *upstream* portion focuses on the beginning stages of the pandemic - studying its emergence, how to diagnose and detect it, how to prevent its spread either by enforcing certain policies, or by developing temporary treatments. In summary, the major focus of the upstream section is the virus itself and the “here and now” aspects. The *downstream* portion focuses more on the longer run or planning horizon and requires more fundamental knowledge and data about the virus. Some key questions for the downstream portion include: (i) how to predict the transmission dynamics of the virus and forecast the number of cases or demand on resources, (ii) how and when to distribute critical resources, how to develop decision and policy support tools to mitigate the effects of the pandemic, (iii) how to plan for the vaccine and its distribution in a timely manner, and (iv) how to determine when and in what ways life can return to normal.
(iii) *Aspects with Direct vs. Indirect Relationship to COVID-19*. Some studies can be classified as directly related to COVID-19, while others are indirectly related (Fig. 6). *Directly related* studies consider the virus as the “center-piece”. For example, the optimal treatment of COVID-19 patients, vaccine development and distribution, and COVID-19 transmission dynamics. *Indirectly related* studies, on the other hand, consider the *effects* of COVID-19 on various aspects of life, for example, on supply chain, transportation planning, electricity demand, local businesses, and optimal strategies used to mitigate the effects of the virus.
(iv) *Compartmentalized Perspective*. This framework is arguably the simplest and easiest to understand, since it divides or groups studies related to the virus by a specific topic, or “compartments” (Fig. 7). The list of topics can be endless, since it is limited only by people’s imagination, interest, and research. Some examples of “compartments” in optimization in the context of COVID-19 include prediction, detection, effects on supply chain, vaccine distribution, the genome of COVID-19, protocols for cleaning work areas, telehealth improvement, treatment of cancer patients during COVID-19, and utilization of AI and machine learning for classifying CT images.

In summary, one must realize that these frameworks all contain similar, even overlapping, components - the key difference though is how we organized them and from what perspective, or lens, we looked at them. One benefit of these frameworks is that they provide perspectives on topics where there has been a lot of focus and where there is a gap. For example, considering the framework in Fig. 5, it is evident that the majority of the literature so far has covered COVID-19 optimization-related topics in the *early stages* portion. Fig. 3 shows that there have been fewer studies conducted in the areas of *mitigation* and *decision support* (compared to *prediction*, for example) in relation to COVID-19 optimization. This insight could be valuable for researchers to identify areas that need to be studied further. Another benefit these frameworks provide is a structured visualization of key areas that might need more attention (and which can be optimized) when the world is fighting a pandemic, such as COVID-19, and how these areas might relate to each other. Finally, a subtle, yet important insight these frameworks provide is not only the connections that can be drawn between the different *aspects* of the pandemic, but also between their corresponding *optimization models*. These connections can serve as the backbone or framework of a full-scale, all-encompassing decision support system that will guide the management of future pandemics. From common practice, it is evident that creating such a model requires a structured approach and must build upon some skeleton, which is precisely how our frameworks would have the greatest impact. As we will see in the following sections, a model and system of such breadth has yet to be developed. Considering the broad scope of the COVID-19 pandemic, we decided to focus on the topics that fell under the umbrella of prediction and control, or if looking from the frameworks’ perspective, our paper covers studies that fall into the overlap of the *macroscale (public health)*, *later stages (downstream)*, and *directly related to COVID-19* perspectives. Specifically, these topics include optimization of COVID-19 screening testing strategies, prediction models, pandemic prevention and control policies and decisions, resource allocation, and mass vaccination.

The remainder of this section will take a deep dive into the respective topics related to COVID-19 optimization that we have considered. In each subsection below, we present an overview and analysis of the existing literature that has been published in that area.

### A. Screening Testing Strategies

This section covers papers wherein optimization methods were used in relation to COVID-19 screening testing strategies - i.e., pooled testing strategies and sample size, prioritizing population groups to test first, and allocation of testing kits.

Before diving into this section, we would like to differentiate between the terms “screening testing” and “diagnostic testing” [75], [76]. The goal of screening tests is to identify the spread of a disease in a *population*, and it aligns with the *public health* focus; while the goal of diagnostic (or clinical) tests is to detect and then treat a disease within an *individual*, and it aligns with the *medical* focus. Although there have been multiple studies focusing on the optimization of *diagnostic* testing of COVID-19 (using chest X-rays or CT scans – see, e.g., [77]–[80]), they will not be reviewed in this section, since they do not fall within the scope of this paper.

Screening is an extremely important tool for detecting and preventing any further spread of COVID-19, especially when a vaccine is not widely available. If people are able to detect the presence of COVID-19, especially if asymptomatic, they are able to immediately implement mitigation strategies, such as quarantining. However, there is not a simple “one size fits all” approach in optimizing the testing strategies. The available literature proposes different testing strategies to account for the limited stockpile of testing kits available, depending on the severity of COVID-19 in a specific region, while seeking to maximize the detection of the virus.

In instances where testing must be conducted in massive numbers, pooling tests have been used [81]–[86]. This type of testing is designed to estimate the “best” pool size for testing under uncertainty.

Regarding testing strategy, a way to implement testing given constraints on resources and varying infection rates by region is critical to accurately identify clusters and waves. For example, [87] addressed the need for an optimized testing strategy for college and university campuses. The study uses a decision tree analysis to assess various testing strategies, including testing of either just symptomatic or all students. Determining the optimal number of students to test is critical in mitigating any spread of the virus on a campus, as also seen in [85], where an optimization model was developed to estimate the optimal number. In terms of utilizing testing to determine mitigation strategies, [88] found a parallel relationship between increasing the number of tests with increasing numbers of those isolated in Italy. In a different study [89], also involving Italy, an optimization model was constructed and solved to determine how much diagnostic testing would need to be conducted per region in order to maximize COVID-19 infection detection capabilities. Increased swab testing was found, in this instance, to have an inverse relationship with any burden on the hospital system. Finally, [90] used a multi-armed bandit approach to determine the optimal distribution of testing resources considering their limited availability and prioritize which groups in the population receive the test. The study [90] presents the effectiveness of different prioritization policies that allow for the maximum detection rate while minimizing the amount of testing resources used.

There have been several approaches aimed at increasing testing efforts. For example, in China, scientists have focused on testing optimization by increasing the laboratory’s COVID-19 nucleic acid testing capacity [91]. In the United States, a data-driven optimization model was fabricated to estimate which pharmacies should offer testing to accelerate the accessibility of testing. The model found that with facility location optimization efforts, people’s “willingness-to-travel” and receive testing would increase to 94% [92]. In conjunction with testing accessibility, testing number optimization is also a critical variable in mitigating the further spread of the virus while also identifying potential clusters of those infected. Determining a methodology for sampling a particular and optimal number of people would minimize the number of testing kits required and the time of testing [93]. As the literature suggests, testing accessibility by determining an optimal number of testing per group will reduce the number of testing kits needed and the time it takes to test a cluster of potentially infected people. In addition, these optimization measures would assist decision-makers in ensuring that lockdowns would be imposed on a need-basis (ensuring that the reproduction number is less than 1) to account for the potential economic damages that may incur due to prolonged lockdowns [94].

Overall, in the realm of screening testing optimization, the key studies have focused on optimizing testing strategies, specifically pooling tests, determining optimal testing sample sizes, and accessibility. However, we did not find any papers regarding optimizing (using formal methods) the design of testing kits or methodologies.

### B. Prediction

This section covers papers where optimization was used in relation to the prediction of COVID-19 cases, deaths, and hospitalizations, as well as forecasting the demand for critical hospital resources needed to treat COVID-19 patients.

From the start of the pandemic, the ability to forecast the spread of COVID-19 has been crucial for both public and hospital administrators. A good prediction can help with timely planning of critical resources to treat COVID-19 patients. It can also enable the implementation of appropriate mitigation policies (such as lockdowns) in a timely manner to minimize the spread of the virus and avoid reaching hospital capacities.

Regarding the optimization of COVID-19 prediction models, three main approaches have been reported in the literature. The first uses the SEIR (Susceptible - Exposed - Infectious - Recovered) model (or its derivatives) as its basis and applies machine learning and optimization methods to determine the epidemiological parameters of the model [6]–[13], [95]–[106]. The second approach uses a population-based model to simulate the transmission of the virus [14], [15]. Finally, the last approach is purely a machine learning based model. For this type of approach, a data-driven machine learning model is developed to forecast the case count, where the inputs are COVID-19 historical time series data or relevant predictors, and optimization algorithms are used to tune the hyperparameters of the machine learning model [16]–[22].

The SEIR model is an epidemiological compartmental model that has been used to model many infectious diseases, including COVID-19 [23]. The main compartments cover the susceptible, exposed, infectious, and recovered population categories, while variations of the SEIR model usually include some additional compartments (e.g., number of deaths, hospitalized, quarantined, infected but asymptomatic) [107]. Due to the novelty of the virus, its epidemiological parameters are unknown, so the SEIR model is fitted to historical COVID-19 data, and the resulting estimated parameters are used to predict future cases. Bayesian optimization [6], metaheuristics (e.g., particle swarm optimization, stochastic fractal search) [7]–[10], [104], [108]–[114], neural networks [11], [115], [116], and nonlinear curve-fitting based optimization methods [12], [13], [117]–[119] are some of the most popular approaches used to fit the model to the data and estimate the epidemiological parameters of the model, such as the reproduction number. In addition to forecasting COVID-19 cases, some studies considered additional aspects, such as the effect of different non-pharmaceutical intervention policies (social distancing and lockdown) and re-opening plans [101], [114], [120]–[127]. For example, Ghamizi *et al.* [11], in addition to predicting cases and deaths, also developed a model to search for optimal exit strategies - i.e., best policies to re-open from lockdown, while maintaining low infection rates. Here, the problem is formulated as a multi-objective optimization problem and solved by a genetic algorithm, wherein the objective function is to maximize the economic aspect of returning to normal life while minimizing the number of deaths and hospitalizations. Russo *et al.* [10] were interested in determining “day 0” for the outbreak of COVID-19 in Lombardy, Italy, as well as the number of asymptomatic patients, since both of these factors affect the length and rate of virus transmission. Guan *et al.* [13] evaluated the effects of complete or partial lockdown in France.

Other studies simulated the transmission of the virus via population- or agent-based models [14], [15], [127]–[129] and network models [130]. These models typically divide the population into the SEIR compartments, and then employ machine learning techniques and heuristic algorithms to optimize the hyperparameters of the model. Additional parameters or factors that may be related to the transmission dynamics were considered as well, such as population density (and therefore the effectiveness of social distancing) [14], [15], or climate related factors [15].

Finally, some studies [16]–[22], [131]–[146] followed a model-agnostic approach and relied solely on the historical time series data of COVID-19 cases or other relevant predictors to forecast future cases. These methods employ machine learning models (neural networks [17]–[21], [133], [135], [138], [142] and deep learning [139]) to make predictions while using various optimization algorithms (such as Gaussian process regression [16], Bayesian optimization [17], and metaheuristic algorithms [18]–[22], [144], [147]–[149]) to optimize the model hyperparameters. When the only input was COVID-19 time series data, the optimization model was essentially a curve-fitting problem, where the objective function was to minimize the squared error between the predicted and actual values. Other machine learning models considered additional data as inputs, such as clinical data [24] or meteorological factors [150], to predict COVID-19 cases or deaths. This class of papers did not only focus on predicting future cases and deaths - some studies went beyond, or refined their predictions. For example, Sun *et al.* [18] estimated the demand for medical resources, such as PPE, medical personnel, ventilators, ICU beds, and oxygen. Schwab *et al.* [24] trained a machine learning model to predict the likelihood of a patient receiving a positive COVID-19 test, requiring hospitalization, or requiring intensive care. Since these predictions were based on the patients’ clinical data, the authors in [24] also suggested which clinical features (such as demographic or blood analysis measurements) were most predictive for each outcome.

Overall, several approaches have been used to predict the spread of the virus, namely, SEIR-based and machine learning models, all of which require *data* to be built and verified. This aspect is particularly troubling during the initial stages of the pandemic, when no data is available. Also, while these models are reported with very small error margins, it is important to recognize that all these models were based on some assumptions and (potentially incomplete) data such that a different set of assumptions and data could lead to significant changes in outputs.

### C. Prevention and Control: Curbing the Spread and Mitigating the Effects

This section covers papers where optimization methods were used in relation to COVID-19 prevention and control - i.e., social distancing, mitigation efforts, and quarantining upon infection.

As the pandemic continues to take lives and a toll on healthcare and the economy, decision makers are faced with the challenge of controlling the spread, preventing additional surges, and determining the best exit strategies. A key question is how to return to normalcy and revive the economy without sparking another wave of COVID-19 cases and overwhelming hospitals. Optimal control problem formulations have been a popular way of modeling and optimizing mitigation strategies and assessing their effect on the spread of the virus [98], [151]–[159]. These frameworks are based on the SEIR model and utilize reinforcement learning (RL) [152], [154] and optimization approaches [153], [160]–[162] to determine the optimal exit strategies. The exit strategies range from cyclic short-term lockdowns to gradual release policies, where different groups of people are allowed to resume normal life activities over staggered time periods. The objective is to minimize the adverse effects of the pandemic on the economy without overwhelming hospital capacities. For example, Kompella *et al.* [154] used an agent-based pandemic simulation approach to model interactions between people in conjunction with an RL model to optimize mitigation policies. This model also accounts for imperfect information, such as false test results and inconsistent adherence to non-pharmaceutical interventions.

Metaheuristic optimization approaches have also been applied to solve the optimal control strategies of the pandemic. These strategies range from the implementation of social distancing to reach herd immunity [163], [164], to increasing testing and quarantine requirements [165], and to developing traditional Chinese medicine (TCM) prevention programs [166]. In [165], the authors developed a nature-inspired model to simulate the distribution process of COVID-19 in different countries and strive to maximize the number of “safe” countries (those that are immune to COVID-19). Each country can be categorized as safe, safe but susceptible, infected and can transmit, and infected but cannot transmit the virus; and countries can help each other by increasing testing capacity, enforcing quarantine measures, and tracking infected individuals that may travel between countries and spread the virus. All these actions are designed to control the pandemic and minimize the risk of infected individuals transmitting the virus to others. In [166], the authors acknowledged the benefits of TCM in treating COVID-19 patients, but realized the need to create diversified prevention and treatment programs for different groups of community residents. TCM is a system of treatment plans or programs that are mainly based on herbal medicine, and one of its principles states that treatment must be individualized, there is no “one-size-fits-all” solution. Recognizing the impossibility of creating an individual TCM treatment plan for each person, the authors targeted groups of people. A fuzzy clustering method was used to divide the population into groups, and a metaheuristic approach (water wave optimization) was developed to optimize the different TCM programs under resource constraints.

Finally, classical optimization approaches have been used to control the spread of COVID-19. Stochastic optimization (e.g., [167]) and game theory approaches (e.g., [168]) were implemented to determine the optimal timing and duration of social distancing policies, and other mathematical programming models were developed to determine both personal and mass protection strategies [169], as well as targeted immunization models [170]. In [171], the authors created a linear programming model to study the trade-off between the expected fatality rate due to COVID-19 and the return to normal activities. Deaths were minimized by optimizing the implementation of non-pharmaceutical interventions, such as social distancing and mask mandates. In [172], the authors developed a decision tool to determine the optimal timing and duration of physical distancing (PD) interventions. The objective is to maximize a measure of control over the pandemic by implementing PD while minimizing the deaths due to COVID-19 and economic costs (which are reflected by the duration of the PD interventions). Finally, Brandao [173] formulated a calculus of variation approach to analyze complex scenarios with competing factors and limited resources. In the context of COVID-19, the competing factors are health and economy, and the optimal control policies require a balance between the two.

In summary, the optimization of mitigation policies is integral to the prevention and control of the COVID-19 virus, especially considering its highly transmissible and oftentimes invisible (i.e., asymptomatic) nature. These studies focused on developing models that would suggest an optimal prevention or mitigation strategy or provide insight into the effects and consequences of different strategies. Studies approached the problem from different angles, with various assumptions, model parameters, and considerations, which implies that no study has considered *all* aspects or has approached the problem from a holistic perspective. Additionally, for multi-objective studies, the two key competing objectives were minimizing cases and maximizing economic benefit, and it would be interesting to consider other objectives as well.

### D. Resource Allocation and Distribution

This section covers papers in which optimization methods were used in relation to the allocation and distribution of critical resources to care for COVID-19 patients (e.g., ICU wards, ventilators, and PPE).

Due to the COVID-19 pandemic impacting and ravaging the globe, available critical resources were in sudden high demand, causing a need for optimization in resource allocation and distribution to maximize patient survival. Critical resources, for the purposes of this literature review and in the context of COVID-19, include PPE (i.e., masks, gloves, gowns, etc.), ventilators, oxygen, ICU beds, and trained medical personnel. Hospitals in certain areas suffered from faster resource depletion than others, causing some areas to makeshift ICU wards in parking lots, stadiums, and other alternative areas. In this regard, optimization of resources and resource distribution was needed to ensure that areas with higher cases (and thus higher demand) were able to access the necessary resources to minimize the number of deaths due to COVID-19.

As an example, an optimization model was developed based on available data to forecast the trend in virus transmission, therefore allowing decision-makers to prepare for sudden surges in demand and corresponding resource needs [25]. Other optimization models considered the allocation and sharing of specific critical resources, such as ventilators [26], [174] and ICU beds [175]. Additionally, medical supply chain networks [176] and medical resource allocation models have been developed and implemented to optimize resource [177]–[179] or patient transfer [27]. In [27], the most optimal load sharing strategy (transfer of ventilators vs. transfer of patients requiring intensive care) was determined, where the minimized cost function was dependent on the number of ICU units above capacity. Similarly, to account for optimized resource planning, simulation environments that used the synergy of deep learning-based predictions and linear optimization were used to plan for resource allocation due to the demands of the COVID-19 pandemic [28]. Decision models were also developed to assist physicians and hospitalists in making decisions pertaining to the allocation of ICU beds - when areas experience surges in COVID-19 infected patients requiring hospitalization, ICU bed availability dramatically diminishes. To combat surges in infected patients requiring intensive care, binary integer optimization models were developed to model the best allocation, and a Monte-Carlo simulation was used to support the output decision [29]. A data-driven combined genetic algorithm and particle swarm multi-objective optimization method was used to solve for allocation of critical resources [30]. Route planning problems [180], [181] were also considered, where optimization methods were applied to determine the optimal route to the closest hospital.

In addition to ICU beds, ventilators, and other critical care resources, testing was also a high-demand, low-supply resource, particularly during the early stages of the pandemic where vaccinations were unavailable, critical to combatting the pandemic. One study used an integer programming formulation to optimize the number of tests a country could conduct while also producing methods to increase testing capacity [31]. Another study [182] addressed the optimal distribution of rapid diagnostic kits when the demand greatly exceeded the supply. Here, the objective was to minimize the loss function that corresponds to the efficiency of an allocation strategy.

Vaccines are also a critical resource which, due to their premature manufacturability, are also in high demand but low supply. Therefore, an optimized distribution of vaccines is paramount for minimizing the number of deaths associated with COVID-19. One method [32] proposed in the literature uses a time-varying linear optimization-based approach that accounts for various relevant epidemiological variables, such as population density and infected ratios.

In summary, equitable and timely distribution of medical resources when they are scarce is an important measure to combat the spread. The literature has presented studies that have focused on the distribution of key critical resources, such as ICU beds, ventilators, vaccines, and even patients. Similar to studies conducted under other topics, the completeness and accuracy of these models are based on available data, assumptions made, current knowledge of the virus, and computational capabilities, which are all, unfortunately, limited.

### E. Mass Vaccination and Vaccine Distribution

This section covers papers in which optimization methods were used in relation to COVID-19 vaccine distribution and mass vaccination.

Optimization of vaccine allocation and prioritization is critical for achieving herd immunity and returning to a level of pre-pandemic normalcy. In cases of limited vaccinations and resources, vaccine distribution designed to minimize the number of deaths, particularly in vulnerable populations, is essential, especially due to the challenge of getting the vaccine to the population, e.g., third world countries. Thus, the logistics of mass vaccination is an imperative aspect to consider. In this regard, [183] developed a bilinear nonconvex optimization model to determine the best location for vaccination sites. Other studies [184], [185] developed a vaccination drive-through simulation tool to optimize the operation and effectiveness of mass vaccination facilities by implementing an agent-based modeling technique and a machine learning model, respectively. It is important to note that during the time of this literature review, there were options for both single- and two-dose vaccinations. Many COVID-19 vaccines require two doses; however in the realm of limited resource availability, policymakers considered administering only a single dose - in this case, determining the efficacy of a single-dose vaccine would be imperative [186]. In [186], the authors developed a model to determine optimal allocation strategies with various dosage vaccinations to reduce viral spread and transmission. The results suggested that a mix of single- and two-dose vaccination campaigns may be a game changer in containing the pandemic.

In some of the optimization models considered, the variables pertaining to distribution include the allocation prioritization of certain groups (e.g., age, presence of comorbidities), and strategies for vaccine distribution [187], [188]. For example, in Brazil, a group of engineers and scientists sought to develop an optimal strategy for vaccine distribution utilizing real-time data from China [189]. By formulating an inverse problem and using a differential evolution (DE) based optimization algorithm, the parameters of the SIR (Susceptible-Infected-Removed) model were then identified. This study proposed two optimal control problems (single- and multi-objective). The first problem involved minimizing the number of infected individuals during treatment, which utilized a DE algorithm. The second problem sought to minimize both the number of infected individuals and the quantity of vaccine concentrate administered during treatment. Researchers have also developed a data-driven mechanistic model of COVID-19 transmission to find optimal vaccine distribution strategies, specifically in China, to reduce the effects of the virus [190].

Another approach in vaccine allocation optimization is the use of an age-stratified model [191]. Using this approach, an optimal vaccine allocation plan was determined based on three metrics: deaths, symptomatic infections, and hospitalizations.

ICU and non-ICU availability was seen as a key metric to evaluate the implementation of different mitigation techniques. For example, the optimization study found that any vaccine with an efficacy of greater than 50% would be able to slow the pandemic while preventing healthcare systems from becoming overwhelmed. The study also found that a vaccine with an efficacy of greater than 70% would allow for full control and containment of the COVID-19 pandemic. Even with such formal optimization tools available for vaccine allocation, there are still many unknown variables, such as immune response duration, that contribute to the mitigation of the spread of COVID-19. Additional insight about these variables would enable the creation of more accurate models that could account for more refined scenarios, rather than making assumptions that may or may not be entirely valid. Similarly, South Korea also utilized an age-stratified model [192]. With the limited vaccination supply available in South Korea during the time of this study, an age-structured model integrated susceptible, latent, asymptomatic and infectious, symptomatic and infectious, and recovered groups as the epidemiological compartments. A different study assessing COVID-19 vaccine distribution optimization in Quezon City in the Philippines also utilized an age-stratified and quarantine-modified SEIR with nonlinear incidence rates (“ASQ-SEIR-NLIR”) compartmental model [193].

Vaccine allocation optimization studies have also considered vaccine equity and fair allocation of vaccines. In a case study focusing on vaccine distribution in Mexico [194], the authors considered different fairness schemes that guided the allocation and included parameters that described vulnerable groups in the population (in terms of size and risk profiles). This particular study also addressed the need to approach allocation by individual state needs. Inequalities in vaccination distribution are further underscored when resources are scarce, whereas the complexity of vaccine distribution increases when resources are readily available; thus, the literature dedicated to vaccine equity during distribution emphasizes the need to address all scenarios when making decisions geared towards vaccine distribution. In a case study focusing on a developing country [195], the modelers and engineers use an inventory-location based mixed-integer linear programming model, based on the distribution of the influenza vaccine, which incorporates equitable objective functions to show the model’s applicability in a COVID-19 context.

Another aspect of vaccine optimization is pricing. Although the vaccine is freely available to anyone in the United States, there is a cost associated with the development, allocation, and administration of vaccines. Reference [196] utilizes an optimization and game theoretic approach to specifically look at Pfizer-BioNTech and Moderna within the US market. The objective is to minimize the total government cost for the vaccine while ensuring that the public demand is met, and the manufacturers achieve a target profit. The results of this study show that even with high production and distribution costs, it is possible to achieve a win-win situation - low vaccine prices while still meeting the demand and ensuring a target profit for the manufacturers.

Overall, studies related to vaccination optimization have focused on the distribution and fair prioritization of vaccines. A common approach was to use an age-stratified model and account for vaccine efficiency; however, there are more factors that can be accounted for in the models to provide more realistic results. In the studies considered, limited and accurate data availability and knowledge about the virus and vaccines are key aspects that hinder the development of accurate models.

### F. Decision Support Tools

This section covers papers in which optimization methods were used in relation to developing decision support tools to combat the spread of COVID-19.

While many studies mention the potential of their models to aid in decision-making processes (e.g., [11], [94], [172], [197]), few actually fit our definition of a decision support tool. For the purpose of this paper, we define a *decision support tool* as a model or tool that utilizes formal optimization methods and explicitly suggests optimal action plan(s). As such, models that utilize decision analysis techniques (such as the analytic hierarchy process (AHP) or are based on utility theory) were not considered.

Several studies have developed decision support systems in relation to various aspects of the pandemic, mainly resource allocation, patient treatment, and non-pharmaceutical intervention (NPI) strategies.

Most resource allocation models can be viewed as decision support tools, since they do suggest a plan for the optimal distribution of resources. For example, [29] and [30] considered an optimal allocation of ICU beds and PPE, respectively, given the scarcity of available resources. However, besides medical devices and equipment, other resources to consider include medical staff (nurses, doctors, technicians), hospital bed availability, and the physical locations of hospitals or emergency medical vehicles. In [33], the authors developed a decision support system for scheduling shifts of physicians. Due to increased hospital workloads, it is important to ensure that both COVID-19 patients and regular patients receive appropriate care while considering the physical and mental well-being of the healthcare workers. Thus, a mixed-integer programming model was proposed to optimize the shift scheduling of physicians. The objective of the model is to minimize the exposure of physicians to the virus while maintaining healthcare service operations at a satisfactory level. In addition to the availability of medical staff, it is important to consider healthcare facility capacities and readiness to accept new patients. To optimize the admission process, the authors in [198] formulated a multi-objective problem using a Pareto-Optimization based algorithm, where the model chooses the most suitable hospital for the patient (based on hospital readiness level and the patient’s condition) with the least admission time. Studies [34], [35], [199] considered the problem of selecting the optimal location for temporary hospitals and medical vehicles. [34] recognizes the fluctuation of incoming patients to a hospital and the resulting stress it inflicts on the hospital system, both resource-wise and financially. To mitigate the undesirable burdens due to the dynamic inflow of patients, the authors developed a model that utilizes a Gray-based decision support framework to select the best location for a temporary hospital for COVID-19 patients. Similarly, [35] models the optimal placement of emergency medical vehicles, specifically for the case when refugee camps are nearby. Due to the high-density populations in refugee camps, a fast response time is critical to avoid spikes when a case has been identified. To ensure that a medical response team can isolate an infected refugee person as quickly as possible, while not neglecting the regular demand load for their services, the authors have developed a spatial hypercube queuing model to determine the optimal locations for emergency medical vehicles.

Other studies have developed decision tools that pertain to decisions regarding patients’ or people’s behaviors (NPIs). For example, [36] utilized a machine learning approach using Bayesian optimization to determine the medical needs and survivability of COVID-19 infected patients. Thus, this model helps hospital workers decide whether a patient would require hospitalization, ICU bed, ventilator, or oxygen, based on their current condition, demographics, and existing comorbidities. Decision tools that provide optimal NPI strategies as outputs are another subset to look at. Some of these models have been mentioned in previous sections (e.g., [11], [154], [172]), while [37] demonstrates how an evolutionary surrogate-assisted prescription (ESP) AI model can be used to determine the most optimal intervention strategies. The surrogate model approach enables the generation of a large number of candidate NPI solutions. Each of the solutions can then be evaluated with respect to balancing the need to curb the spread of the pandemic and minimizing the economic impact.

As shown in the examples above, decision support tools play a major role in managing the pandemic and mitigating its effects. Reference [200] suggests that modeling efforts are applicable to various areas of decision-making in relation to the pandemic, such as decisions regarding disease transmission, resource management, and public healthcare. Although there has been some work done in this area to date, we have not found any decision support systems that can capture a broader scope of the pandemic effects - e.g., no studies have considered incorporating prediction, resource allocation, and prevention capabilities in a single model.

## V. Discussions: Lessons Learned and Gaps

The literature review revealed numerous optimization methods and models developed to assist decision makers in a COVID-19 pandemic context, while considering various epidemiological parameters and public health variables. The literature makes it clear that combating the virus requires effective implementation of mitigation strategies and policies aimed at minimizing infection and transmission rates. It can be argued that optimization serves a critical role for decision makers by instilling some level of confidence prior to implementing mitigation strategies. Carefully tailored models and decision support tools, such as epidemiological models (i.e., SEIR) for forecasting, and models for resource allocation can assist decision makers in preventing the drainage of critical supplies and minimizing the number of cases and deaths associated with COVID-19.

With a plethora of studies presented in the literature, there is a common drawback, or limitation, to the models across all domains - the lack of sufficient, unified data and knowledge about the virus. This limitation is arguably the main root of the current gaps, namely, unstable or inconsistent model accuracy and reliability, very low to non-existent level of implementation of the developed models on a broader (population-wide) scale, and the limited scope of individual models that do not account for a holistic perspective of the virus. Also, all of the reported models are based on some assumptions that may be true (but not guaranteed) at a given moment yet may fail as time goes on and the circumstances change. Moreover, a vast majority of the models is data-driven, i.e., rely on existing data to tune the model parameters - obviously during the early stages of the pandemic, when no data is available, these models become inapplicable. Clearly, the complexity of the virus, its variant, how it transmits, and affects our lifestyle is beyond our current modeling and computational capabilities.

Ideally, it would be of great use to have a “digital twin” model of the pandemic, which would account for all the nuances of the virus, its effects, and its multidisciplinary nature. The frameworks presented in this paper could serve as the foundational structure for such a model. For example, consider the *Macroscale vs Microscale* perspective – on the micro-level, a mechanistic (e.g., epidemiological) model based on the virus microbiology and bio-behavior would be of great value to understand the nature and dynamics of the virus, particularly in the beginning stages when data is scarce; on the macro-level, a data-driven model may be more appropriate to determine, for example, the optimal resource allocation plan or re-opening policy. From this example it is obvious that not only is it important to view the problem from different perspectives, but also consider integrating different modeling approaches. Mechanistic- and data-driven models could complement each other and possibly be combined as well. Such a combined model would have to reflect the holistic perspective rather than the individual aspects (such as prediction, resource allocation, or prevention) of the pandemic. As more data and relevant information becomes accessible and available, the potential to optimize and improve existing models and tools, which can aid in determining optimal intervention policies and mitigation strategies, increases.

Although the literature reviewed overwhelmingly addressed COVID-19, many of the developed optimization models have not actually been implemented or utilized, and the added value which can be achieved from the implemented models has not been quantified. For example, although age-stratified and other models that seek to prioritize vaccine distribution in a manner that would minimize the number of deaths and burden on the healthcare system exist, many countries did not apply strategies recommended by the developed optimization models. Data and model reliability and accuracy may have been a variable in implementation hesitation. Instilling trust in decision makers requires an accurate and adaptable model. However, the novelty of the virus and its variants, and the lack of sufficient or accurate data pose a great challenge for the verification of existing models. Unfortunately, since the models reported in the literature have not gained widespread popularity among decision makers, many decisions were ad hoc and based on expert knowledge and opinion. In many situations “optimal” prevention and control strategies were determined by trial and error. This approach is very subjective, which could be detrimental, especially considering the global, complex, and dynamic nature of the virus. Moreover, the lack of a systematic approach may annul all previous efforts to control the spread and leave uncertainty about their effectiveness. Table 2 provides some examples of regulations and COVID-19 statistics in a few countries as of June 30, 2021. From the frameworks’ perspective, the four regulations shown in Table 2 fall into the prevention, testing, and vaccination compartments. Without the framework or models, these regulations appear to be implemented almost randomly, and we can see the resulting effects on cases, deaths and vaccination status are inconclusive. For example, if we create a utility function for each regulation, where stricter regulations and greater accessibility to testing and vaccinations are defined by a greater utility value, then the respective total utilities summarized in Table 2 describe the overall “goodness” of the regulations. The USA and India both have high utility values (16 and 17, respectively) compared to Spain (12), yet the number of cases is 10 times greater in the first two countries (USA and India). Australia on the other hand has the lowest case count, as shown in Table 2, despite the mediocre level of regulations and very low vaccination rate. This analysis shows how critical it is to have a structured framework and model to assess and improve the effectiveness of regulations and other decisions, as well as ensure that no aspects get ignored.

**TABLE 2:** Examples of COVID-19 Response Actions in Different Countries^*^

Country	Regulations^4^					% Fully Vaccinated	Confirmed Cases	Deaths
	Stay-at-home	Face coverings	Testing policy	Vaccination	Utility			
USA	Recommended	Required in all public spaces	Open public testing	Universal	16	47.2%	33,664,991	604,598
Brazil	Required (except essentials)	Required outside-the-home	Symptomatic & key groups	Vulnerable + some others	15	12.4%	18,557,141	518,066
Australia	Required (except essentials)	Required in all public spaces	Anyone with symptoms	Vulnerable + some others	15	5.9%	30,643	910
China	Required (except essentials)	Required in some public spaces	Open public testing	Universal	16	89%	103,769	4,847
India	Required (few exceptions)	Required outside-the-home	Anyone with symptoms	Vulnerable + some others	17	4.2%	30,411,634	399,459
Saudi Arabia	No measures	Required in all public spaces	Open public testing	Universal	15	4.6%	487,592	7,819
Egypt	No measures	Required outside-the-home	Anyone with symptoms	All vulnerable	13	0.8%	281,282	16,169
Italy	Required (except essentials)	Required in some public spaces	Open public testing	Universal	16	31.3%	4,259,909	127,566
Spain	Recommended	Required in some public spaces	Anyone with symptoms	All vulnerable	12	38.3%	3,808,960	80,875

^*^ Data from Johns Hopkins University, for January 22, 2020 – June 30, 2021^5^

^4^ https://ourworldindata.org/policy-responses-covid

^5^ https://doi.org/10.1016/S1473-3099(20)30120-1

Finally, the complexity of the virus and its unprecedented effects create unanticipated challenges when attempting to develop and update models. This pandemic is a multi-player, multi-objective, and multi-disciplinary problem, which is why it has been approached by parts, rather than as a whole. For example, some studies focused on optimizing prediction models of COVID-19 cases, while others focused on equitable allocation of vaccines in a certain country. An additional challenge and level of complexity are introduced by the nonuniform and detached nature of the developed models - different sets of assumptions, varying approaches, parameters, and objectives. Unifying and standardizing these models would be ideal; however, the complexity of the processes would grow exponentially and may become computationally intractable. Utilizing a modular approach that could eventually be assembled together based on the frameworks presented in this paper could be a possible direction. Developing individual models that could be integrated into a main system model would require a fundamental framework that could account for the relationships between the subsystems, and a uniform approach and overarching common goal.

To summarize, data availability, accessibility, and accuracy are prevalent issues. Countries use non-standardized approaches when collecting and interpolating relevant data. In addition, some nations have hindered efforts for COVID-19 data accessibility. Moreover, unfortunately, political and economic incentives have led some governments to intentionally provide false reports on the status of COVID-19. Therefore, the detection of trends and the ability to develop and verify a data-driven model becomes inaccurate, which directly affects decision makers. The development of a reasonably robust algorithm where such intricate data is not required but does not compromise the accuracy of the output prediction, could be a possible future direction.

COVID-19 has proven to be a ubiquitous phenomenon that has affected all areas and components of life. Through this literature review, the critical need for data accessibility and accuracy to support data-driven decision-making when creating mitigation strategies, is apparent. The development of proper models and decision support tools for future pandemics can assist in ensuring that the healthcare system is well equipped for another pandemic and that the number of lives lost is minimal.

## VI. Conclusion

As mentioned at the outset, this is the first paper that has provided a review of optimization studies in the context of COVID-19 with a focus on prediction and control. From the literature reviewed, it is evident that researchers, scientists, and decision-makers are constantly attempting to develop and determine the most effective and optimal way to combat the spread of COVID-19 and its effects on the general population. The utilization and implementation of optimization techniques has been a powerful tool for studying and analyzing the potential impacts of the virus, as well as the effectiveness of certain mitigation strategies. For example, models that optimize the prediction of virus transmission, forecast demand for hospital resources, or provide exit strategies can aid decision makers in determining the best course of action and understanding its implications.

This literature review focused on a subset of optimization studies related to the COVID-19 pandemic. Specifically, studies related to the optimization of prediction models of COVID-19 cases and deaths, forecasting the demand of critical hospital supplies to treat patients, optimization of screening testing strategies, resource allocation, vaccine distribution, and the development of decision support tools were considered. Four new frameworks were introduced to facilitate the structure of different perspectives on COVID-19 and other imminent pandemics. Additionally, this literature review sought to identify any gaps in the current literature.

Assessing the literature using the four proposed frameworks underscored the need for better optimization models to minimize the external and internal effects of a novel and ubiquitous pandemic, such as COVID-19. The frameworks can be used to account for various aspects of the virus that are all intertwined with one another, while simultaneously being dependent on the rate of community transmission and hospitalizations. For example, the microscale versus macroscale framework can be used to gain a macro-perspective behavior of the virus while being heavily dependent on its performance at a microscale level.

Finally, in terms of future research directions, there are still many gaps and challenges remaining due to the ongoing nature of the COVID-19 pandemic. For example, a better understanding of the virus and its transmissibility, though challenging due to its evolutionary nature, would allow for more accurate and effective mitigation and resource allocation optimization efforts. Also, better accessibility, uniformity and accuracy of data would provide vastly more robust and reliable mechanistic models, particularly when dealing with virus variants that continue to mutate and dampen mitigation efforts. Regarding vaccinations and the impact of mass vaccinations, due to the ongoing efforts to vaccinate the general population, there is still a lack of data and information on vaccine distribution and formulation optimization. As COVID-19 continues to evolve, prediction and forecasting have become powerful tools in preparing and protecting decision-makers, hospitals, supply chains, governments, and civilians in the realm of testing, prevention and control, vaccination, and resource allocation. However, these tools, which are designed to provide more accurate and effective optimization strategies, can only become useful if data are accessible and trustworthy.
